# Complexity Quantification of Driving Scenarios with Dynamic Evolution Characteristics

**DOI:** 10.3390/e26121033

**Published:** 2024-11-29

**Authors:** Tianyue Liu, Cong Wang, Ziqiao Yin, Zhilong Mi, Xiya Xiong, Binghui Guo

**Affiliations:** 1 School of Artificial Intelligence, Beihang University, Beijing 100191, China; liutianyue@buaa.edu.cn (T.L.); zb2342112@buaa.edu.cn (C.W.); yinziqiao@buaa.edu.cn (Z.Y.); mizhilong9@buaa.edu.cn (Z.M.); xiongxiya@buaa.edu.cn (X.X.); 2 Zhongguancun Laboratory, Beijing 100094, China; 3 Beijing Advanced Innovation Center for Future Blockchain and Privacy Computing, Beijing 100091, China; 4 LMIB, NLSDE, Beihang University, Beijing 100091, China

**Keywords:** autonomous vehicles, driving scenario complexity, safety assessment, complexity quantification

## Abstract

Complexity is a key measure of driving scenario significance for scenario-based autonomous driving tests. However, current methods for quantifying scenario complexity primarily focus on static scenes rather than dynamic scenarios and fail to represent the dynamic evolution of scenarios. Autonomous vehicle performance may vary significantly across scenarios with different dynamic changes. This paper proposes the Dynamic Scenario Complexity Quantification (DSCQ) method for autonomous driving, which integrates the effects of the environment, road conditions, and dynamic entities in traffic on complexity. Additionally, it introduces Dynamic Effect Entropy to measure uncertainty arising from scenario evolution. Using the real-world DENSE dataset, we demonstrate that the proposed method more accurately quantifies real scenario complexity with dynamic evolution. Although certain scenes may appear less complex, their significant dynamic changes over time are captured by our proposed method but overlooked by conventional approaches. The correlation between scenario complexity and object detection algorithm performance further proves the effectiveness of the method. DSCQ quantifies driving scenario complexity across both spatial and temporal scales, filling the gap of existing methods that only consider spatial complexity. This approach shows the potential to enhance AV safety testing efficiency in varied and evolving scenarios.

## 1. Introduction

Autonomous Vehicles (AVs) have garnered widespread attention owing to their potential to enhance the transportation systems safety and deliver substantial economic and environmental benefits. In recent years, AVs have seen considerable technological advancements alongside the development of deep learning. However, no SAE level 4 [1] autonomous vehicles have been commercially deployed to date, primarily due to the incomplete verification of their safety [2]. One of the key steps in the development and deployment of AVs is conducting comprehensive safety assurance testing [3]. Common methods for AV testing include Naturalistic Field Operational Tests (N-FOT) [4] based on mileage and scenario-based testing. Due to the repetitive and simple scenarios in natural driving environments, significant events such as accidents are rare. Thus, N-FOTs require the accumulation of hundreds of millions of miles in order to demonstrate AV safety [5], leading to high time and economic costs. To accelerate the safety testing of AVs, researchers are exploring scenario-based testing methods that intentionally generate critical scenarios to increase the probability of their occurrence [6,7,8,9,10]. The complexity of a scenario is a key indicator that defines the importance of critical scenarios [11]. It is recommended that scenarios with higher complexity be prioritized for testing to improve testing efficiency [12,13].

For autonomous vehicles, complex traffic environments impact the entire driving process, including perception, decision-making, and control. In [14,15], the authors indicated that the performance of AV algorithms is often inversely correlated with the complexity of the scenarios they encounter. When assessing AVs, test results across different complexity scenarios may not be directly comparable [16]. For instance, an AV scoring 95 in a simple scenario might only score 50 in a complex one, whereas another AV scoring 85 in a simple scenario could achieve 70 in a complex one. Such score discrepancies inadequately reflect the overall performance of AVs. Therefore, quantifying the complexity of driving scenarios is crucial for AV safety assurance testing.

Several studies have analyzed the complexity of driving environments from the perspective of drivers. In [17,18,19], the authors investigated the relationship between workload, driver mental load, and complexity of the traffic environment. In [20,21], experienced drivers directly annotated the complexity of diverse driving scenarios through video clips. Nevertheless, these subjective methods are limited by the low number of scenarios, and are challenging to scale up for large datasets. Moreover, the impact of model quantification results on AV intelligent algorithms remains unexplored.

In addition, some objective quantification methods based on environmental factors have defined scenario complexity using variables such as traffic density [22], weather conditions, and road hazards [23], among others. For example, a dynamic driving environment complexity quantification method was proposed in [11] by describing spatial and temporal interactions among vehicles from the perspectives of quantity, variety, and relationships. However, this approach ignores static traffic elements and other traffic participants. To supplement this, static traffic elements such as driveable area size, weather visibility, and road friction coefficient were integrated in [6] to comprehensively quantify testing environment complexity. In [24], the scenario complexity was divided into two parts for evaluation, namely the road semantic complexity and traffic element complexity. In [19], the authors considered dynamic factors such as the pedestrian density, cyclist density, and number of vehicles passing through per unit time. In [13,25], a tree structure model of factors influencing intelligent driving systems was devised by combining expert evaluation methods to determine the respective contributions of each factor to the scene complexity.

Existing studies on methods of driving scenario complexity quantification are summarized in Table 1. In summary, the existing studies primarily focus on analyzing the impact of scenario elements by categorizing them into static and dynamic factors. However, several issues remain unresolved. First, an inadequacy exists in the analysis of correlation between scene factors. The stronger the correlation between factors, the higher the redundancy in their reflected evaluation information, necessitating appropriate adjustments to their contributions to scene complexity. Second, the term ’dynamic’ mentioned in the aforementioned studies refers to relatively dynamic elements within the environment, but fails to consider the dynamic evolutionary property of scenarios themselves.

In the context of this paper, the standard definition in ISO 34501 [26] is adhered to and reiterated: the **Scene** is the snapshot of all entities, including the environment and both static and dynamic entities, all actors and observer self-representations, and the relationships between these entities. The **Scenario** is the sequence of scenes which describes a time span; a scenario is a description of the evolution of a series of scenes over time. According to this definition, the complexity models established in existing research are oriented towards scenes rather than scenarios, and consequently overlook the variability of elements within the traffic environment. The performance of AVs in scene-oriented settings does not necessarily guarantee consistent performance in scenarios characterized by significant fluctuations in the constituent elements of the scene.

**Figure 1:**
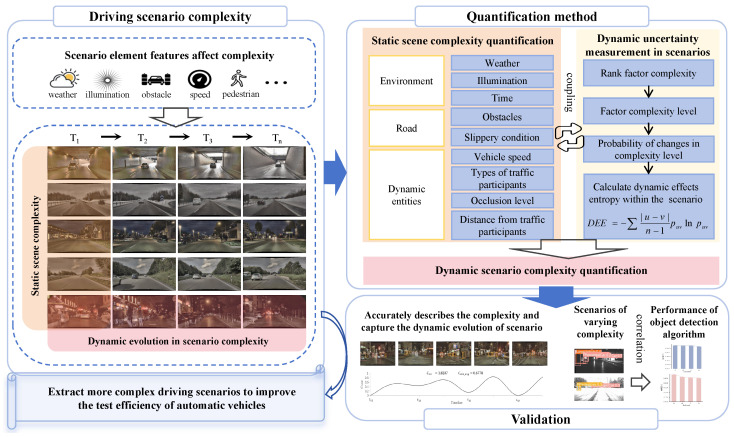
Illustration of the quantification method for dynamic driving scenario complexity. The scene is the snapshot of all entities, while the scenario is the sequence of scenes which describes a time span [26]. Scenario complexity is influenced by elements and features such as weather, illumination, and obstacles, and their dynamic evolution further increases scenario uncertainty. This study develops a scenario complexity quantification method incorporating both static features and dynamic evolution. In this context, we investigate the effects of different complexity levels on object detection performance.

Therefore, this paper proposes the Static Scene Complexity Quantification (SSCQ) method, which comprehensively considers the impacts of environmental conditions, road conditions, and dynamic entities on scene complexity. Additionally, it analyzes the correlation among factors within each dimension. On this basis, the information entropy [27] is introduced to measure the dynamic changes of scene elements, thereby quantifying the uncertainty added to scenarios due to changes in complexity. Consequently, the Dynamic Scenario Complexity Quantification (DSCQ) method is proposed. Furthermore, we validate the quantification results by correlating them with the performance of object detection algorithms. Figure 1 shows the flow of this work. Notably, this quantification framework is flexible and extensible, allowing for the inclusion of additional evaluation dimensions and indicators in the future.

## 2. Proposed Method

The demand for higher capabilities in autonomous driving systems escalates when faced with more complex driving scenarios. Consequently, a model that effectively characterizes the complexity of driving scenarios is crucial for testing and evaluating AVs. As mentioned above, existing complexity quantification methods are oriented towards static driving scenes rather than dynamic driving scenarios, and fail to account for the intricacies arising from the temporal variation of scene elements. In this study, we propose a comprehensive quantification method of scenario complexity on both the temporal and spatial scales. In Section 2.1, we refer to the way that [6,24] categorized the factors influencing scene complexity into static and dynamic elements and further subdivide these factors into three dimensions. Subsequently, in Section 2.2 we quantify the static scene complexity based on the partitioned dimensions. Finally, we measure the uncertainties caused by variations in these factors over the observation period in Section 2.3 to quantify the complexity of dynamic scenarios.

### 2.1. Factors Influencing Driving Scene and Scenario Complexity

**Figure 2:**
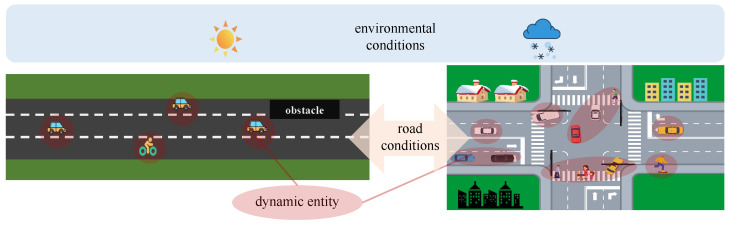
Domains of interest for different quantification dimensions.

The complexity of driving scenes is influenced by three key dimensions: natural environmental conditions, road conditions, and dynamic entities in driving scenes. The complexities of these three dimensions are denoted as $C_{1}$, $C_{2}$, and $C_{3}$, respectively. The domains of influence of these dimensions are depicted in Figure 2, which provides detailed information on influencing factors, notation, and the corresponding values and complexity indices. For continuous variables, the complexity indices are directly determined based on their values or by establishing a functional relationship with the complexity. For discrete variables, complexity indices are assigned on a scale of 0 to 1, reflecting their impact on overall complexity. Specifically, the complexity indices for Weather are ranked based on the complexity of different weather conditions as outlined in [28], while the indices for Time and Types of Traffic Participants are directly adopted from [6,13], respectively.

(1) Natural Environmental Conditions Complexity ($C_{1}$): The complexity of environmental conditions is defined according to the weather, illumination, and time of day of the scene in which the tested AV operates. Specific weather conditions can range from clear skies to heavy rain or fog. Illumination refers to the available light intensity, which varies from best conditions for computer vision to overall dark situations. Daytime and nighttime present different lighting conditions and levels of visibility, each contributing differently to the complexity of the driving environment.

(2) Road Conditions Complexity ($C_{2}$): The complexity of road conditions is defined according to the slippery condition of the AV’s drivable path and the quantity of obstacles. Slippery road surfaces caused by rain, ice, or snow contribute to complexity, directly impacting vehicle safety and maneuverability. Additionally, the number of obstacles increases driving difficulty, with a higher obstacle count indicating a more complex environment that necessitates more precise perception and planning.

(3) Dynamic Entities Complexity ($C_{3}$): This dimension considers dynamic entities in the driving environment, encompassing the following factors:

Ego Vehicle’s Speed: The speed of the ego vehicle affects the frequency of interactions with surrounding dynamic entities and the available reaction time; at higher speeds, the need for quicker decision-making and faster responses escalates, thereby augmenting complexity.

Types of Traffic Participants (TPs): Different types of TPs, such as cars, bicycles, and pedestrians, each have different behavior patterns and interaction modes, which imposes various requirements on the AV’s perception and decision-making system; for example, pedestrian behavior is more random, while cars follow traffic rules.

Occlusion Levels: The degree to which other vehicles or objects are obscured; high levels of occlusion mean that the ego vehicle’s sensors have a limited field of view, requiring stronger prediction and reasoning capabilities to handle potential sudden situations.

Distances to Other TPs: The distance between the ego vehicle and other traffic participants is crucial for safety and decision-making, with shorter distances requiring higher precision and faster response to prevent collisions and ensure safety.

The complexity quantified in this study is relative, and the proposed complexity quantification method offers a general framework that is adaptable to various datasets. Given the inconsistencies in annotation scope and content across autonomous driving datasets, it is challenging to provide universally consistent quantification standards for all datasets. However, as the proposed method does not rely on the specific annotation ranges of any particular dataset, researchers can apply this method to rank and assign complexity indices to scenario factors in other datasets. Additionally, the proposed framework supports flexible expansion or removal of factors within the three complexity dimensions, ensuring adaptability to diverse datasets and scenarios.

### 2.2. Static Scene Complexity Quantification

The complexity of static driving scenes is collectively determined by the complexities $C_{1}$, $C_{2}$, and $C_{3}$ of the three dimensions mentioned above, which can be described as follows:

(1) $$C_{sne}=\left(C_{1}+C_{2}\right)*C_{3}.$$

The complexity calculations for $C_{1}$ and $C_{2}$ are as follows:

(2) $$C_{1}=\left(\omega_{11}x_{11}+\omega_{12}x_{12}+\omega_{13}x_{13}\right),$$

(3) $$C_{2}=\left(\omega_{21}x_{21}+\omega_{22}x_{22}\right).$$

Initially, $\omega_{ij}$ represents the weights of the $j^{th}$ influence factor within the $i^{th}$ dimension, and is set equally with ${\bar{\omega }}_{ij}$. Then, these weights are adjusted based on the conflict analysis of factor *p*. Specifically, a stronger correlation between factor *p* and other factors *q* indicates a higher degree of redundancy in that factor’s reflected evaluative information and a correspondingly weaker level of conflict. This can undermine the evaluative strength of the indicator, necessitating a decrease in the weight assigned to factor *p*. The degree of conflict between factor *p* and other factors is denoted by Equation (4), where $r_{pq}$ represents the Pearson correlation coefficient between influence factors *p* and *q*, while the adjusted weight is presented as Equation (5):

(4) $$R_{p}=\sum_{q}\left(1-r_{pq}\right),$$

(5) $$\omega_{ij}=\left({\bar{\omega }}_{ij}+\frac{R_{j}}{\sum_{j}R_{j}}\right)/2.$$

**Figure 3:**
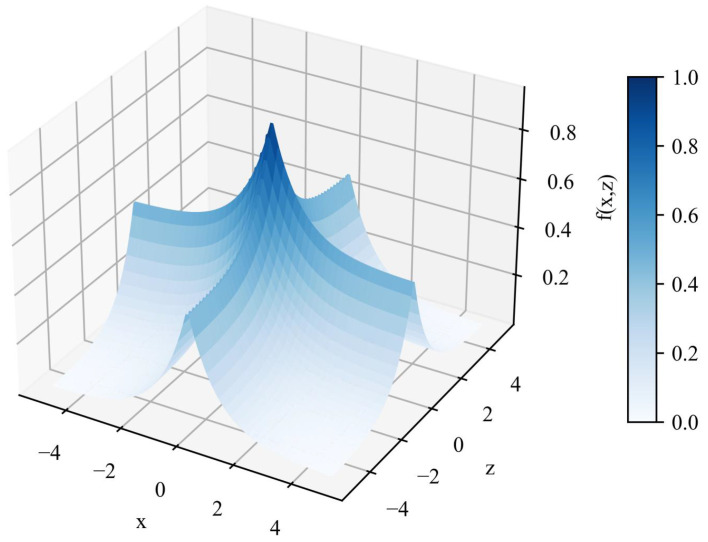
Relationship between complexity and distance to other traffic participants.

The complexity $C_{3}$ is calculated using Equation (6), where β is the scale factor and *m* denotes the TP number:

(6) $$C_{3}=\beta *V_{ego}*\sum_{j=1}^{m}f\left(x,z\right)*\ln\left(1+e^{x_{type}}\right)*\ln\left(1+e^{x_{occ}}\right),$$

(7) $$f\left(x,z\right)=0.5e^{-|x|}+0.5e^{-|z|},$$

where *x* and *z* represent the horizontal and vertical distances between TPs and the ego vehicle, respectively. The relationship between *x*, *z*, and complexity is illustrated in Figure 3.

### 2.3. Dynamic Scenario Complexity Quantification

In the process of driving, the complexity of the scene is constantly varying, leading to scenarios that are both dynamic and uncertain. For instance, in the case of high traffic density, the speed of surrounding vehicles may frequently adjust, and the complexity introduced by such dynamic variability will increase the difficulty of driving decision-making. Similarly, during the process of entering or exiting a tunnel, rapid changes in illumination can further elevate scenario complexity, affecting the accuracy of target recognition by cameras. Consequently, it is crucial to develop a scenario complexity quantification model that can adapt to these dynamic evolutionary property in order to effectively address emerging complexity.

The complexity indices corresponding to the varying values of influencing factors in Table 2 are sequenced to ascertain their respective complexity levels. It is noteworthy that the complexity level associated with an influence factor may change at successive time points. Table 3 outlines the probability of changes in complexity level; the rows represent the complexity levels of a factor at time *t*, the columns represent these levels at the subsequent time t+1, and $P_{u,v}$ denotes the probability of a transition in complexity level from *u* to *v* between two adjacent time points within a specified time frame.

Based on the respective probabilities of changes in complexity levels within the scenario, the information entropy is utilized to quantify the uncertainty arising from the complexity variations of each factor [27], as illustrated in Equation (8). Entropy is a classical theoretical tool for describing system uncertainty that is widely applied in complex systems to capture the impact of dynamic variations on system states. In dynamic driving scenarios, complexity primarily arises from the evolving scenario factors and the uncertainty that they introduce. Entropy provides a natural theoretical foundation for quantifying this complexity. Previous studies such as [11,25] have employed entropy to analyze the contributions of static and dynamic factors to scene complexity. However, these applications predominantly focused on localized effects of specific factors, and lacked systematic characterization of the overall dynamic evolution of scenario complexity.

Furthermore, because the complexity contributed by different degrees of change varies, a weighting coefficient is introduced to measure these varying degrees. The weighting coefficient is the ratio of the change in complexity level to the range of the complexity level of that factor. As shown in Table 3, the complexity level of a factor ranges from 1 to n and the change in complexity level ranging from *t* to t+1 is u−v; then, the weight coefficient is (u−v)/(n−1). We define this as the Dynamic Effect Entropy (DEE) based on the weighted information entropy obtained from Equation (8) and the degree of change in the impact factor, which we then use to measure the dynamic effect of scenario complexity, as shown in Equation (9). DEE quantifies the impact of the uncertainty due to dynamic changes in the investigated factor’s complexity on the complexity of the scenario over the observation period *T*. The design of DEE builds upon entropy theory by incorporating the unique characteristics of dynamic driving scenarios. DEE employs a weighting mechanism to quantify how variations in complexity contribute to overall scenario uncertainty. While its computation depends on inputs such as illumination or vehicle speed, the computational logic is consistently based on the entropy-based measurement of dynamic changes, with weighting then applied to their magnitudes. Although the computational process can be flexibly adjusted to accommodate different input parameters, the formalized model remains unchanged, offering a universal analytical framework for quantifying the complexity of dynamic scenario.

(8) $$H_{uv}=-\sum p_{uv}\ln p_{uv}$$

(9) $$DEE=-\sum \frac{\left|u-v\right|}{n-1}p_{uv}\ln p_{uv}$$

The DEE values of each factor are calculated and summed in three dimensions to derive $DEE_{1}$, $DEE_{2}$, and $DEE_{3}$. These values are then utilized to determine the complexity of the dynamic scenario, as illustrated in Equation (10). $\bar{C_{1}}$, $\bar{C_{2}}$, and $\bar{C_{3}}$ correspond to the respectively average values of $C_{1}$, $C_{2}$, and $C_{3}$ over the designated time period.

(10) $$C_{sio}=\left[\left(1+DEE_{1}\right)\bar{C_{1}}+\left(1+DEE_{2}\right)\bar{C_{2}}\right]\left(1+DEE_{3}\right)\bar{C_{3}}$$

## 3. Experiment and Results

### 3.1. Performance Comparison of DSCQ and SSCQ Methods

#### 3.1.1. Dataset

The dataset used in this work was DENSE [29], which covers 10,000 km and contains diverse weather and lighting, including severe conditions such as snow, rain and fog. Its comprehensive annotations and varied testing trajectories make it exceptionally suitable for conducting scenario complexity assessment. To facilitate the continuous measurement of scenario complexity, the dataset was segmented into independent scenario units at every 60 s. The choice of scenario unit length was guided by the need to balance the effective capture of both dynamic changes and computational efficiency. Short intervals may fail to adequately reflect the dynamic evolution of a scenario, while longer intervals might obscure critical short-term variations, weakening the ability to evaluate autonomous driving performance. For practicality, an interval of 60 s was chosen in order to ensure a balance between capturing major dynamic changes and maintaining moderate computational cost. This interval can be flexibly adjusted to suit different application scenarios. Such changes affect only the numerical results without altering the core logic of the DSCQ method which quantifies complexity based on the dynamic changes in the scenario. Data that could not be categorized into any scenario unit were excluded, resulting in a final selection of 9916 frames of data constituting 519 scenario units.

#### 3.1.2. Quantification of Driving Scene and Scenario Complexity

With the above data, the SSCQ method was employed to conduct an initial evaluation of scene complexity. The findings indicated that approximately 30.58% of scenes exhibited complexity between 0 and 0.01 and that 50.60% fell within the range of 0 to 0.1, suggesting that most driving scenes possessed a very low level of complexity. Subsequently, the dynamic effects within each scenario unit were calculated based on the DEE to obtain the quantified scenario complexity ($C_{sio}$) results. The calculation process is shown in Algorithm 1. Among the 519 analyzed driving scenarios, 13.1% exhibited complexity levels between 0 and 0.1. The summarized descriptive statistics of the complexity quantification results are presented in Table 4.
**Algorithm 1:** Driving Scenario Complexity Quantification
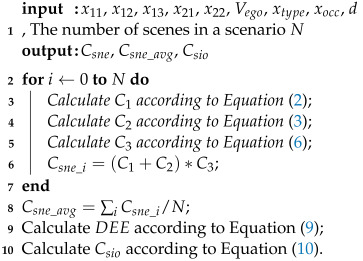


#### 3.1.3. Comparison of Quantitative Results for Scenario Complexity Using DSCQ and SSCQ

**Figure 4:**
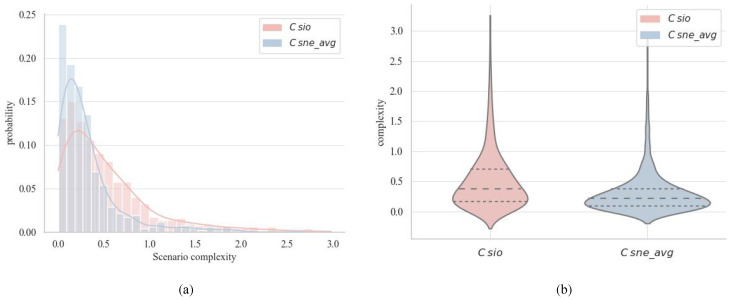
Comparison of quantified scenario complexity results for the DSCQ and SSCQ approaches: (**a**) probability distribution of scenario complexity and (**b**) violin plot of complexity, with dashed lines representing quartiles and width of the plot indicating probability density.

To further demonstrate DSCQ’s ability to capture the effect of dynamic changes on scenario complexity, the average scene complexity within each scenario unit, denoted as $C_{sne\_avg}$, was calculated and compared against the quantification results of $C_{sio}$. The probability distribution and violin plot depicted in Figure 4 illustrates that $C_{sio}$ and $C_{sne\_avg}$ exhibit similar overall trends in the quantification of scenario complexity, with the majority of scenarios having relatively low complexity. However, the quantification results of SSCQ tend to cluster more towards lower complexity levels compared to DSCQ, potentially overlooking numerous highly intricate scenarios characterized by significant dynamic fluctuations. In comparison, $C_{sio}$ adapts to the dynamic evolution of scenarios and identifies a broader range of complex scenarios.

**Figure 5:**
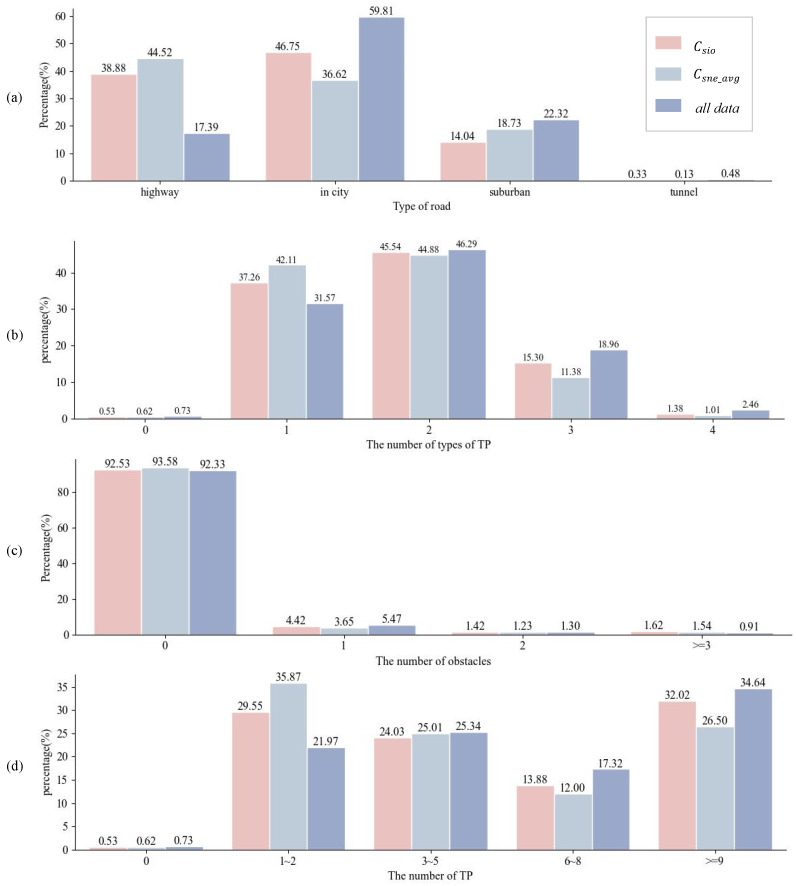
Comparison of scene elements in the top 20% of scenario complexity for $C_{sne\_avg}$ and $C_{sio}$: (**a**) percentage of different road types, (**b**) percentage of number of traffic participant types, (**c**) percentage of obstacle quantities, and (**d**) percentage of traffic participant quantities.

Attention to complex scenarios is paramount in both automated driving testing and real-world driving scenarios. Consequently, this study focuses on comparing the top 20% of scenarios ranked by complexity using the SSCQ and DSCQ methods. Analyzing the composition of road types within complex scenarios, as depicted in Figure 5a, the results from the SSCQ method reveal that highway-type scenarios dominate the set of complex scenarios, primarily due to their high vehicle speeds, which increase scene complexity. Highways typically exhibit relatively consistent road structures, including straight segments, ramps, and exits, and have relatively sparse pedestrian and non-motorized vehicle traffic. Consequently, driving scenarios on highways tend to be more stable, resulting in lower complexity at the dynamic variation level.

In contrast, the DSCQ method predominantly identifies complex scenarios within city areas, aligning with the diversity and uncertainty of urban transportation environments. Urban road networks are inherently more intricate, including intersections, roundabouts, narrow streets, and intensified traffic flows. In addition, diverse behaviors of pedestrians and non-motorized vehicles contribute to the heightened uncertainty in urban driving scenarios. Hence, driving scenarios within city areas exhibit greater instability and complexity when considering dynamic variation. Moreover, the DSCQ model captures the heightened complexity of tunnel scenarios stemming from rapid variations in lighting conditions, traffic flow, and obstacles. DSCQ further indicates that scenarios with more obstacles and a higher number and variety of participants exhibit greater variability and uncertainty, leading to increased scenario complexity. In summary, the complex scenarios identified by DSCQ are more representative of real-world driving environments.

### 3.2. Validity Verification of DSCQ

#### 3.2.1. Case Study

**Figure 6:**
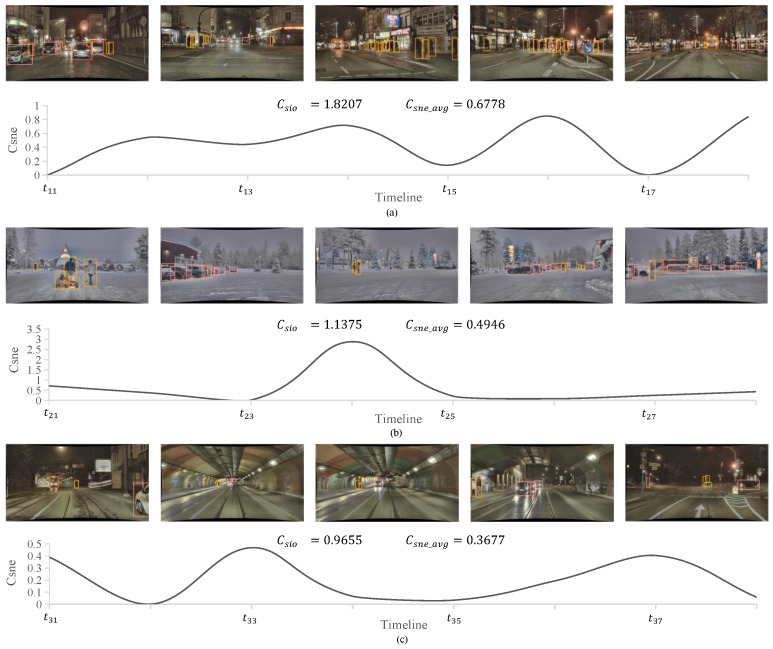
Examples of driving scenes and corresponding quantified scene complexity and scenario complexity results. The curve graphs illustrate the fluctuations in $C_{sne}$ within the sample scenario. The $C_{sio}$ and $C_{sne\_avg}$ of the scenario are calculated to demonstrate the discrepancy between DSCQ and SSCQ in quantifying scenario complexity. The results indicate that although the example scenario exhibits relatively low scene complexity, there are significant fluctuations in complexity within the scenario. This increase in uncertainty is captured by DSCQ but not by SSCQ.

To further demonstrate the feasibility of the proposed complexity quantification methods and delineate the distinctions between the two quantification models, we selected three scenarios with pronounced complexity disparities as examples and investigated them using both models for illustrative purpose. These scenarios contain various driving conditions such as time, road type, weather, etc., and dynamic changes. The curve graphs in Figure 6 illustrate the fluctuations in $C_{sne}$ within the sample scenario. Finally, several general conclusions regarding the quantitative model of driving scenario complexity are derived.

In the first scenario (Figure 6a), $C_{sne\_avg}$ = 0.6778 and $C_{sio}$ = 1.8207. Despite the complexity of each scene in this scenario not being particularly high, its location within a bustling district amplifies uncertainty. The lighting conditions, number of obstacles, and vehicle speeds undergo frequent fluctuations; particularly notable are the significant variations in pedestrian volume. These factors collectively elevate the scenario’s complexity.

The second scenario (Figure 6b), with $C_{sne\_avg}$ = 0.4946 and $C_{sio}$ = 1.1375, is situated within an irregular road environment which lacks clear regulatory constraints. The variability in the number of obstacles is significant, and the behaviors of traffic participants are more diverse. For example, children may play on the ground, posing a risk of unexpected entry into the driving path. These factors contribute to increased complexity, placing higher demands on the perception and decision-making capabilities of autonomous driving systems.

The third scenario Figure 6c, with $C_{sne\_avg}$ = 0.3677 and $C_{sio}$ = 0.9655, involves the process of traversing a tunnel. Within the tunnel, frequent changes in lighting conditions can compromise the reliability of sensor data. Additionally, there can be discrepancies in traffic flow and vehicle speeds between the inside and outside of the tunnel, necessitating timely adjustments in strategies by the autonomous driving system in order to adapt to the evolving road conditions. Consequently, the need to traverse the tunnel introduces greater complexity to the driving scenario.

Based on the above cases, the following conclusions can be drawn:

(1) The SSCQ method can accurately describe the complexity of different driving scenes.

(2) The DSCQ method, on top of measuring the complexity of driving scenes, also quantifies the uncertainty arising from dynamic changes within the scenario, allowing it to capture complex scenarios that SSCQ may overlook.

(3) While certain driving scenes may not be inherently complex, fluctuations in the scene parameters can increase the complexity of the associated scenarios.

#### 3.2.2. Analysis of the Impact of Scenario Complexity on Object Detection Performance

The core tasks of an autonomous driving system encompass perception, decision-making, and control. The accuracy of perception is fundamental to ensuring the safety and effectiveness of the entire system, as only through precise object recognition can the system make correct decisions and execute appropriate control measures [30]. Compared to other sensors, cameras offer higher accuracy in object detection and lower costs [31], making them indispensable sensors in AVs. This section aims to explore the correlation between scenario complexity and the object detection algorithm performance of gated cameras. Through experimental analysis of various complex scenarios, we aspire to uncover how gated cameras perform under different environmental complexities.

- Experimental Design and Algorithm

The experimental data are detailed in Table 5. To ensure that the experimental results were not influenced by the amount of training data, we sorted 519 scenarios in ascending order based on the complexity quantified by the DSCQ method and divided them into four equally sized subsets with complexity levels increasing from A to D. We conducted object detection on these four subsets with different complexity levels and compared the detection performance across the groups. The YOLOv5 model was chosen for its advantages in inference speed, detection accuracy, and small file size, which have made it widely applied in autonomous driving object detection tasks.

- Implementation Details

We utilized the Pytorch framework for model construction. The software environment utilized CUDA v12.2, cuDNN v7.6.5, and Python 3. For the training process, we employed the pretrained yolov5s.pt model with an original image size of 1280 × 720 pixels. The training and validation image sizes were uniformly scaled to 640 × 640 pixels, and the hyperparameters were set as follows: training epochs = 10, batch size = 16, confidence threshold = 0.25, optimizer = stochastic gradient descent (SGD), and learning rate = 0.01.

- Evaluation Metrics

To evaluate the accuracy of the algorithm, we employed the mean average precision (mAP) [32] as the primary metric, specifically using mAP@0.5 and mAP@0.5:0.95; mAP@0.5 denotes the average precision across all categories at an IoU threshold of 0.5, while mAP@0.5:0.95 provides a comprehensive assessment by considering the average precision across a range of IoU thresholds from 0.5 to 0.95 with a step size of 0.05. The formula for calculating mAP is as follows:

(11) $$mAP=\frac{1}{K}\sum_{i=1}^{K}\int_{0}^{1}P_{i}\left(R_{i}\right)dR_{i}$$

where *K* is the number of categories, while $P_{i}$ and $R_{i}$ represent the precision and recall of category i, respectively, and are calculated as follows:

(12) $$P=\frac{TP}{TP+FP}$$

(13) $$R=\frac{TP}{TP+FN}$$

where TP denotes the number of true positive predictions, FP denotes the number of false positive predictions, and FN denotes the number of false negative predictions.

Furthermore, we compared the confidence of the object detection results across the four subsets. In the YOLO model, for each detected object, the confidence score reflects the model’s certainty that the object is within the specified bounding box and belongs to a particular class. This score is a combination of the probability of an object being present in the bounding box and the probability distribution over all classes. Confidence scores provide a measure of uncertainty, with higher confidence scores suggesting that the model is more certain about the presence and class of the detected object.

- Results

**Figure 7:**
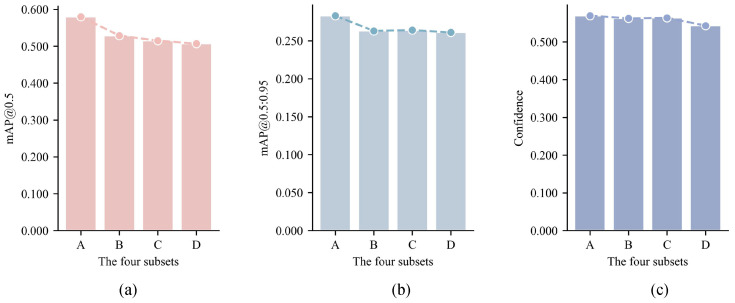
Performance comparison of object detection in different subsets: (**a**) comparison of mAP@0.5, (**b**) comparison of mAP@0.5:0.95, and (**c**) comparison of average confidence.

The experimental results presented in Figure 7 demonstrate a general decline in detection accuracy and confidence as the complexity level increases from subset A to subset D. Notably, the detection results for subsets B and C are relatively close together, which may be attributed to the small difference in scenario complexity between these two subsets.

Based on these observations, it can be inferred that scenario complexity has a certain impact on object detection performance; as the scenario complexity increases, the detection accuracy tends to decrease and the uncertainty becomes stronger. Hence, the effectiveness of the DSCQ method proposed in this study for quantifying the complexity of driving scenarios is validated through these findings.

## 4. Conclusions

This paper has proposed the DSCQ method for quantifying the complexity of driving scenarios, aiming to fill the existing research gap in measuring the complexity and uncertainty of dynamic driving scenarios. The proposed method evaluates driving scenario complexity by integrating static scene complexity and the uncertainty arising from dynamic changes. Specifically, static scene complexity examines the impact on complexity of different environmental conditions, road conditions, and dynamic entities in the traffic environment. Meanwhile, the uncertainty analysis of dynamic changes explores the evolving properties of scene elements in order to quantify the informational upsurge associated with their variations.

To validate the model’s effectiveness in quantifying scenario complexity, experiments were conducted on real traffic data. Scenario complexity was measured using both the DSCQ model, which considers spatiotemporal evolution characteristics, and the SSCQ model, which disregards these dynamics. Three different types of scenarios were used as case studies to demonstrate the ability of DSCQ to quantify dynamic scenario complexity. Our results underscore the superior ability of DSCQ to precisely capture dynamic uncertainties, making the extracted complex scenarios more representative of reality. Furthermore, a preliminary exploration into the influence of scenario complexity on object detection performance was undertaken. Our findings reveal a decrease in both accuracy and confidence of object detection in more complex scenarios. In conclusion, our research results demonstrate that the proposed quantification method not only provides an accurate description of the complexity differences in various driving scenarios but also reveals the variations in scenario complexity stemming from different degrees of dynamic evolution in similar scenes.

There are several potential applications for the proposed model. First, by extracting complex scenarios, it allows more attention to be paid to the performance of AVs under challenging circumstances, thereby improving testing efficiency. Second, when considering the diverse performance of autonomous driving algorithms across multiple scenarios, reliance on a single scenario for evaluation may yield biased conclusions that inadequately reflect algorithms’ overall effectiveness and reliability. The model presented in this paper can provide support for building a comprehensive system evaluation for coupled evaluation of AV scenarios and algorithms by quantifying the scenario complexity. Moreover, this quantification method is computationally simple, and is considered suitable for integration into AVs for real-time monitoring of scenario complexity, thereby providing immediate support and assistance for human driver takeover warnings.

Although the object detection algorithm proposed in this study is highly representative, completely eliminating the specificity of analytical conclusions remains challenging. However, our conclusions indicate prospects for analyzing other algorithms related to autonomous driving. Future work could consider using more detailed data annotations in simulation environments to achieve more precise quantification of scenario complexity, and could analyze the performance of decision-planning algorithms in scenarios of varying complexity beyond perception algorithms. Our results indicate that DSCQ already has significant practical application potential, laying a solid foundation for future research into extending complexity analysis to the decision-making and control modules.

## Figures and Tables

**Table 1:** Summary of scene complexity measurement methods.

Category	Study	Scenario Elements	Method
Subjective quantitative methods based on the driver’s perspective	[17]	/	Analyzing the relationship between driver’s mental workload and traffic environment complexity
	[18]	/	Analyzing the relationship between driving workload and road condition complexity
	[19]	/	Analyzing the relationship between driver workload and traffic complexity
	[20]	/	Experienced drivers directly annotated complexity ratings using video clips
	[21]	/	Experienced drivers directly annotated complexity ratings using video clips
Objective quantitative methods based on environmental factors	[11]	Encounter angles (θ), relative speeds (*v*), and relative distances (*d*)	$C=f_{1}\left(\theta \right)\times f_{2}\left(v\right)\times f_{3}\left(d\right)$
	[6]	Drivable area size, weather visibility, road friction coefficient (static scene complexity $C_{S}$), and dynamic traffic elements ($C_{D}$) as described in [11]	$C=\alpha_{S}C_{S}+\alpha_{D}C_{D}$
	[19]	Dynamic factors such as the pedestrian and cyclist density and the number of vehicles passing through per unit time	Combining different parameter values of traffic elements
	[22]	Traffic density	Analyzing the impact of traffic density on takeover performance
	[23]	Road hazards	Analyzing the impact of road hazard events on takeover performance
	[24]	Road semantic complexity ($C_{R}$) and traffic element complexity ($C_{E}$)	$C=\lambda_{1}C_{R}+\lambda_{2}C_{E}$
	[13]	Lighting environments, lane line parameters, road parameters, longitudinal and lateral speed, road congestion	Developing a tree structure model of factors and integrating it with the analytic hierarchy process
	[25]	Obstacles, lanes, traffic lights, identifying and responding to other traffic participants	Utilizing expert evaluation methods combined with information entropy theory

**Table 2:** Factors influencing scene complexity. The complexity index for the Weather factor is assigned uniformly between 0 and 1 based on the complexity order of various weather conditions from [28], while the complexity indices for Time and Types of Traffic Participants are determined according to [6,13], respectively.

	Influence Factor	Notation	Value	Complexity Index
**Environment**	Weather [28]	$x_{11}$	Clear	0
			Rainy	0.25
			Light fog	0.50
			Snow	0.75
			Dense fog	1
	Illumination	$x_{12}$	Best computer vision weather	0
			Low dynamic range	0.33
			High dynamic range	0.66
			Overall dark	1
	Time [13]	$x_{13}$	Day	0
			Night	1
**Road**	Obstacles	$x_{21}$	0	0
			1	0.33
			2	0.66
			3+	1
	Slippery condition	$x_{22}$	Dry	0
			Wet	0.33
			Slushy	0.66
			Full snow coverage	1
**Dynamic entities**	Vehicle speed	$V_{ego}$		
	Types of traffic participants [6]	$x_{type}$	Pedestrian	0.7
			Ridable vehicle	0.8
			Passenger car	0.9
			Large vehicle	1
	Occlusion level	$x_{occ}$	No occlusion	0
			>10%	0.1
			>40%	0.4
			>80%	1
	Distance from traffic participants	(x,z)		f(x,z)

**Table 3:** Probability of changes in complexity level.

Complexity Level	1	2	⋯	v	⋯	n
**1**	$p_{11}$	$p_{12}$	⋯	$p_{1v}$	⋯	$p_{1n}$
**2**	$p_{21}$	$p_{22}$	⋯	$p_{2v}$	⋯	$p_{2n}$
⋯	⋯	⋯	⋯	⋯	⋯	⋯
**u**	$p_{u1}$	$p_{u2}$	⋯	$p_{uv}$	⋯	$p_{un}$
⋯	⋯	⋯	⋯	⋯	⋯	⋯
**n**	$p_{n1}$	$p_{n2}$	⋯	$p_{nv}$	⋯	$p_{nn}$

**Table 4:** Summarized descriptive statistics of the complexity quantification results.

	Number of Records	Mean	Std.Dev.	Min.	Median	Max.
$C_{sne}$	9916	0.3116	0.4992	0	0.0928	4.2423
$C_{sne\_avg}$	519	0.3086	0.3418	0	0.2133	2.6916
$C_{sio}$	519	0.5163	0.4907	0	0.3809	2.9796

**Table 5:** Scenario complexity-based dataset partitioning.

Complexity Range	Subset	Average of Scenario Complexity
0–25%	A	0.082
25–50%	B	0.2689
50–75%	C	0.5281
75–100%	D	1.1911

## Data Availability

The dataset used in this study is available at: https://ieeexplore.ieee.org/document/9157107, accessed on 21 October 2024.
